# Charcot–Marie–Tooth causing HSPB1 mutations increase Cdk5-mediated phosphorylation of neurofilaments

**DOI:** 10.1007/s00401-013-1133-6

**Published:** 2013-06-01

**Authors:** Anne Holmgren, Delphine Bouhy, Vicky De Winter, Bob Asselbergh, Jean-Pierre Timmermans, Joy Irobi, Vincent Timmerman

**Affiliations:** 1 Department of Molecular Genetics, VIB and University of Antwerp, 2610 Antwerpen, Belgium; 2 Laboratory of Neurogenetics, Institute Born-Bunge, 2610 Antwerpen, Belgium; 3 Laboratory of Cell Biology and Histology, Department of Veterinary Sciences, University of Antwerp, 2020 Antwerpen, Belgium; 4 Peripheral Neuropathy Group, VIB Department of Molecular Genetics, University of Antwerp, Universiteitsplein 1, 2610 Antwerpen, Belgium

**Keywords:** Charcot–Marie–Tooth disease, Peripheral neuropathy, Small heat shock protein, Neurofilaments, Cyclin-dependent kinase

## Abstract

Mutations in the small heat shock protein HSPB1 (HSP27) are a cause of axonal Charcot–Marie–Tooth neuropathy (CMT2F) and distal hereditary motor neuropathy. To better understand the effect of mutations in HSPB1 on the neuronal cytoskeleton, we stably transduced neuronal cells with wild-type and mutant HSPB1 and investigated axonal transport of neurofilaments (NFs). We observed that mutant HSPB1 affected the binding of NFs to the anterograde motor protein kinesin, reducing anterograde transport of NFs. These deficits were associated with an increased phosphorylation of NFs and cyclin-dependent kinase Cdk5. As Cdk5 mediates NF phosphorylation, inhibition of Cdk5/p35 restored NF phosphorylation level, as well as NF binding to kinesin in mutant HSPB1 neuronal cells. Altogether, we demonstrate that HSPB1 mutations induce hyperphosphorylation of NFs through Cdk5 and reduce anterograde transport of NFs.

**Electronic supplementary material:**

The online version of this article (doi:10.1007/s00401-013-1133-6) contains supplementary material, which is available to authorized users.

## Introduction

Charcot–Marie–Tooth disease (CMT) is the most common inherited neurodegenerative disorder of the peripheral nervous system. CMT neuropathy is clinically and genetically heterogeneous, caused by mutations in a large number of genes with diverse functions. The genes associated with CMT disease may have a specific role in Schwann cells or in the peripheral neurons. Although some genes are expressed in every tissue and cell, the CMT-causing mutations selectively affect peripheral nerves [46].

Missense mutations in the small heat shock protein HSPB1 (HSP27) are causative of axonal CMT type 2F (CMT2F) and distal hereditary motor neuropathy (HMN) [26]. The HSPB1 protein is ubiquitously expressed and belongs to the family of highly conserved ATP-independent molecular chaperones [28, 34]. We recently reported that some HSPB1 mutations show a higher chaperone activity than the wild-type protein, affect microtubule dynamics and impede axonal transport [5, 7, 22]. However, the pleiotropic nature of the HSPB1 protein suggests that some neuron-specific functions could contribute to peripheral neuropathy. Indeed, CMT-causing mutations in HSPB1 have been shown to cause aggregation of the neurofilament light protein, and therefore disruption of the neurofilament (NF) network [26, 74].

Neurofilament light (NFL), together with neurofilament medium (NFM), neurofilament heavy (NFH) and α-internexin, are neuron-specific proteins that form the main cytoskeletal elements in mature neurons [73]. As their highest concentration is found along the axon, disruption of the NF network may underlie degeneration of peripheral nerves. Moreover, NFs in axons are extensively phosphorylated. Phosphorylation of their C-terminal domains modulates their structural dynamics and function [17, 59] and is mainly regulated by the interplay of five proline-directed kinases: p42/44 mitogen-activated protein kinase (ERK1/2 MAPK), p38 MAPK, c-jun-N terminal kinase (JNK), glycogen synthetase kinase 3 (GSK3) and cyclin-dependent kinase 5 (Cdk5) [3, 57, 65]. Each kinase can phosphorylate distinct lysine–serine–proline (KSP) motifs in the C-terminal domains of NFM and NFH, suggesting their ability to differentially affect the functioning of NFs [2, 16]. Here, we studied the effect of mutant HSPB1 on NF dynamics and phosphorylation.

## Materials and methods

### Cell lines and DNA constructs

Human neuroblastoma cells (SH-SY5Y) were cultured in minimum essential medium (Life Technologies, Ledeberg, Belgium) supplemented with 10 % fetal calf serum, 1 % non-essential amino acids (Life Technologies) and 1 % glutamine (Life Technologies) and incubated at 37 °C in a humidified atmosphere (5 % CO_2_). Stable SH-SY5Y cell lines were generated by lentiviral transduction [48] using constructs encoding wild-type (WT) or mutant [Arg127Trp (R127W), Ser135Phe (S135F) and Pro182Leu (P182L)] HSPB1 as previously described [5]. The PAGFP-NFM construct was a generous gift from Prof. Dr. Anthony Brown (Department of Neuroscience, Ohio State University, Columbus, USA) [20]. Cells co-expressing HSPB1-WT and PAGFP-NFM, or HSPB1-P182L and PAGFP-NFM, or PAGFP-NFM alone, were generated for live cell imaging experiments.

Knockdown of Cdk5 was performed with MISSION^®^shRNA Cdk5 constructs (Sigma, Bornem, Belgium). The shRNA constructs were sh3 (TRCN0000195513), sh4 (TRCN0000194974), sh5 (TRCN0000021465), sh6 (TRCN0000021466) and sh7 (TRCN0000021467). The efficiency of these constructs was verified and the level of Cdk5 knockdown determined by Western blotting (WB) (data not shown). One construct (sh3) was selected to create double stable cell lines with the HSPB1 WT and mutant constructs.

### Antibodies and inhibitors

We used the following antibodies: anti-HSPB1 (Enzo Life Sciences, Antwerpen, Belgium), anti-NFL DA2 (Cell Signaling, Danvers, MA, USA), anti-actin (Sigma), anti-Cdk5 DC17 (Santa Cruz, Heidelberg, Germany), anti-phosphorylated NFs SMI34 (Abcam, Cambridge, UK), anti-kinesin heavy chain antibody KN-01 (ab9097) (Abcam), anti-NFM NF-09 (Abcam), anti-phophoY15-Cdk5 (Abcam), anti-phosphorylated NFs SMI-31 (Eurogentec, Seraing, Belgium), anti-non-phosphorylated NFs SMI-33 (Eurogentec), anti-NFH SMI32 (Eurogentec), anti-APP (Sigma), goat anti-mouse IgG1-HRP (SouthernBiotech, Birmingham, Alabama, USA), goat anti-mouse IgG2a-HRP, goat anti-mouse IgG2b-HRP (SouthernBiotech), peroxidase-conjugated goat anti-mouse IgG (Jackson Laboratories Inc., Bar Harbor, Maine, USA), peroxidase-conjugated goat anti-rabbit IgG (Jackson Laboratories Inc.), anti-V5 (Life Technologies), Alexa Fluor^®^ 488 goat anti-mouse IgG (Life Technologies) and Alexa Fluor^®^ 594 goat anti-mouse IgG (Life Technologies).

The specificity of the antibodies against phosphorylated and non-phosphorylated NFs was verified using cell lysates treated with or without phosphatase inhibitor or arctic phosphatase. The SMI-31 antibody is directed against the phophorylated KSP repeats in the C-tail of NFH and to a lesser extent NFM, while the SMI-33 antibody is described to detect non-phosphorylated NFH and to a lesser extent NFM [49, 53, 60]. The arctic phosphatase treatment dephosphorylated NFH as indicated by a shift in electrophoretic mobility on the agarose gel [57]. As expected, the SMI-31 antibody did not probe NFH or NFM after treatment with arctic phosphatase, while the SMI-33 antibody still probed NFH and NFM after arctic phosphatase treatment (data not shown). The phosphorylation status of the NFs was therefore assessed with the SMI-31 antibody. The SMI-34 antibody was used to detect the phosphorylated S/TPXH/K/R repeat motif in the C-domains of NFH and NFM, specifically phosphorylated by Cdk5/p35 [9].

As inhibitors we used: SB203580 (p38 inhibitor, Enzo Life Technologies), SP600125 (JNK inhibitor, Enzo Life Technologies), U0126 (MEK1/2 inhibitor, Cell Signaling), TDZD-8 (GSK3β inhibitor, Sigma) and roscovitine (Cdk5 inhibitor, Cell Signaling). Specific kinase inhibitors were diluted to 10 μM directly in medium and cells were incubated for 16–24 h. Efficiency of all inhibitors was verified by WB by assessing the phosphorylation level of the kinase itself or a known downstream substrate unrelated to NFs (data not shown).

### Neuronal differentiation of SH-SY5Y cells with retinoic acid

The protocol was adapted from Ammer and Schulz, 1994 [8, 25]. Glass Bottom Culture Dishes (35 mm, thickness 1.5, 14 mm) (further referred to as ‘imaging dishes’) or 12-well plates were coated overnight with 0.1 mg/ml poly-l-ornithine (Sigma) at room temperature. Cells were seeded at a density of 10.000/cm^2^ in medium with 1 % non-essential amino acids, 1 % glutamine, 2 % fetal calf serum and 10 μM retinoic acid (Sigma). Differentiation was performed in Neurobasal A medium (Life Technologies) containing 2 % B27 supplement (Life Technologies), 1 % glutamine and 20 μM retinoic acid. The medium was refreshed every other day. After 7 days of differentiation, cells were imaged, collected for WB or fixed in 4 % paraformaldehyde for immunocytochemistry.

### Western blotting

Unless otherwise indicated, cells were lysed in E1A lysis buffer [20 mM HEPES (pH 7.9) with 1 % (vol/vol) NP-40, 250 mM NaCl, 1 mM EDTA, 20 mM β-glycerophosphate, 10 mM NaF, 1 mM Na_3_OVO_4_, 2 mM DTT] and Complete protease inhibitor mixture (Roche Applied Technologies, Vilvoorde, Belgium). Cells were sonicated for 10–30 s on ice using the U50-control sonicator (IkaLabortechnik, Staufen, Germany) and subsequently incubated for 30 min on ice. Protein concentrations were measured using the Bradford protein assay (Bio-Rad, Nazareth, Belgium) and equal amounts of protein extract were prepared for SDS-PAGE by addition of SDS-PAGE sample buffer and heating the samples at 95 °C for 10 min. Proteins were separated on NuPAGE gels (Life Technologies) and transferred to a nitrocellulose membrane (Hybond™-P, Amersham/Pharmacia) using the wet blotting method. After blocking in 5 % milk powder in PBS or TBS, supplemented with 0.1 % (vol/vol) Tween-20, blots were incubated with a primary antibody overnight at 4 °C and 1 h with a secondary horseradish peroxidase-conjugated antibody. Blots were developed using the enhanced chemiluminescence Plus™ detection system (GE healthcare, Diegem, Belgium). In all experiments, except Triton-X 100 experiments, actin was used as loading control.

Band intensity was determined by quantifying the ‘mean pixel gray’ values using ImageJ software (http://rsbweb.nih.gov/ij/) after Gaussian blur and background subtraction on the inverted image. The ‘mean pixel gray’ values were measured in a rectangular region of interest. Band intensities were normalized to β-actin levels by dividing the ‘mean pixel gray’ values of the band of interest by the actin ‘mean pixel gray’ values. Data from multiple experiments are presented as relative quantities by normalizing to the non-transduced control.

### Immunocytochemistry and immunofluorescence microscopy

Immunostaining was performed according to standard protocol. Briefly, cells were fixed in 4 % paraformaldehyde for 30 min and permeabilized with 0.1 % (vol/vol) Triton-X 100 in phosphate-buffered saline (PBS). Blocking was done with 5 % goat serum (Dako, Heverlee, Belgium) for 1 h, primary antibodies were incubated for 1 h and secondary antibodies for 30 min at room temperature. Nucleus staining was done with Hoechst33342 (Invitrogen), after which cells were mounted with Dako fluorescent mounting medium (Dako) and examined with an Axiovert200 fluorescence microscope, equipped with a CCD Axiocam camera and Axiovision software (Carl Zeiss, Zaventem, Belgium). Images were acquired with a 63× Plan-Apochromat objective (1.4NA) and standard fluorescence filters.

Laser scanning confocal microscopy of immunostained samples was performed on a Zeiss LSM700 microscope equipped with a 63× Plan-Apochromat objective (1.4 NA). Image stacks (sampled at Nyquist resolution in XY and Z) were deconvolved using the ImageJ Iterative Deconvolve 3D plugin with theoretically calculated point spread function, created with the ImageJ Diffraction PSF CD plugin.

Superresolution structured illumination microscopy was performed on a Zeiss ELYRA S.1 microscope equipped with a 63× Plan-Apochromat objective (1.4 NA).

Quantification of the number of V5-positive, or NF-aggregate positive cells was performed manually by counting a minimum of 100 cells per experiment for each cell line. Cells were scored visually. For quantification of the mean fluorescence intensity, image fields were selected at random in the transmitted light mode, without exposure to the fluorescence excitation light. All images were captured with the same exposure time. In the soma or in neurites of respectively undifferentiated or differentiated SHSY-5Y cells, fluorescence intensity was quantified by measuring the ‘mean pixel gray’ value in a region with the same dimensions using ImageJ. All quantifications were performed blind for the treatment conditions and in triplicate.

### Axonal transport studies with PAGFP-NFM

Cells were seeded in an imaging dish and differentiated as explained above. The medium was changed right before imaging. All genotypes were imaged on the same day, during 7 days, for which three different differentiations were performed. Image time series were acquired on a Zeiss LSM700 inverted confocal laser scanning microscope equipped with temperature and CO_2_ incubation. Imaging conditions and microscope settings were identical for all time series. Using a Plan-Apochromat 63×/1.4NA objective and laser light excitation at 1 % transmission of a 10 mW 488 nm diode laser, 512 × 512 images were acquired with a pixel dwell time of 3.15 μs and pixel size of 0.198 μm. Image fields containing several neurites were selected in transmitted light mode and, for photoactivation, a region of interest containing a 15 μm long neuritic segment was drawn in the Zeiss Zen 2009 software. Every time series consisted of (1) two pre-photoactivation frames, (2) the photoactivation phase (2 iterations using the 5 mW 405 nm laser at 100 % transmission) and (3) 147 frames at 10.1 s time intervals. To specifically assess the directionality of movement, we counted the number of photoactivated neurofilaments that could unambiguously be discriminated as individual filaments that moved out of the photoactivated zone either anterogradely or retrogradely at high speed. To quantitatively assess movement of NFs in general, the leading edges of filaments moving out of the photoactivated neuritic segment were tracked by using the Manual Tracking plugin of ImageJ and by using the cell body as a reference point. The XY-coordinates of the filament tracks, originating from the ImageJ output file, were used to analyze the tracks in a custom-made script in R (http://www.r-project.org/). Briefly, the displacement of the leading edge between two consecutive frames was used to calculate the speed and directionality at each time point. A filament was considered in pause if the calculated speed was below 0.06 μm/s; this represents a displacement of three pixels during two consecutive time frames that we considered as the precision limit of our analysis. This value is consistent with earlier reports on analysis of NF movement [67]. These raw parameters were used to classify the speeds into different categories and to extract several parameters that describe the movement pattern. For the data that is presented, the mean parameters were calculated for each NF track, which was used to calculate the average and SEM for each genotype. In total, more than 120 time series, consisting of more than 600 tracks and 40,000 time points, were analyzed.

### Co-immunoprecipitation

Cells were lysed in NP-40 buffer (PBS, 0.5 % (vol/vol) Nonidet P-40, 20 mM β-glycerophosphate, 10 mM NaF, 1 mM Na_3_OVO, 2 mM DTT) and Complete protease inhibitor mixture (Roche Applied Science) and left on ice for 30 min [50, 69, 70, 72]. We considered the insolubility of the NF proteins and therefore worked with total cell lysates. No harsh detergent was added to the lysates before IP. Protein measurement was performed to equalize protein concentration from different cell lines before immunoprecipitation (IP). Sepharose 6B (Sigma-Aldrich) and protein G beads (GE Healthcare) were used and the anti-SMI-31 antibody was incubated overnight at 4 °C, after which the beads were pulled down by centrifugation. The beads were repeatedly washed before preparing SDS-polyacrylamide electrophoreses.

### Preparation of Triton-X 100-soluble/-insoluble fractions

Cells were lysed in a 1 % Triton-X 100 buffer (50 mMTris (pH 6.8) with 1 % (vol/vol) Triton-X 100, 5 mM EDTA, 50 μg/ml leupeptin, 1 mM Na_3_OVO_4_, 20 mM β-glycerophosphate, 10 mM NaF) and Complete protease inhibitor mixture (Roche Applied Science), by pipetting up and down and leaving them on ice for 20 min. After centrifugation at 15,000×*g* for 20 min at 4 °C, the supernatant was collected as ‘Triton-X 100-soluble fraction’ and the pellet fraction (‘Triton-X 100-insoluble fraction’) was resuspended in E1A lysis buffer and sonicated for 30 s. On both fractions, protein concentrations were measured and equal concentrations were subjected to WB. The amyloid precursor protein (APP) was used as a positive control to determine if the experiment was accurately executed, as it is supposed to be Triton-X 100 soluble.

### Transmission electron microscopy

Cells were grown on poly-l-ornithine-coated eight-well chambered Permanox slides (Nunc, Lab-Tek) and treated for neuronal differentiation as described above. Cells were fixed in 0.1 M sodium cacodylate-buffered (pH 7.4) 2.5 % glutaraldehyde solution for 2 h at 4 °C. After rinsing (3 × 10 min) in 0.1 M sodium cacodylate-buffered (pH 7.4), 7.5 % saccharose, they were post-fixed in 1 % OsO_4_ solution for 1 h. After dehydration in an ethanol gradient (70 % ethanol for 20 min, 96 % ethanol during 20 min, 100 % ethanol for 2 × 20 min), cells were embedded in EM-bed812. Ultrathin sections were stained with uranyl acetate and lead citrate, and examined in a Tecnai G2 Spirit Bio Twin Microscope (FEI, Eindhoven, The Netherlands) at 100 kV.

### Statistical analysis

For all experiments, results are shown as average ± standard error of the mean (SEM). GraphPad Prism 5 software was used for statistical calculations. One-way ANOVA with post hoc Bonferroni’s multiple comparison test was used for statistical analysis; *p* values are indicated.

## Results

### Mutation in HSPB1 reduces anterograde axonal transport of neurofilaments

**Fig. 1:**
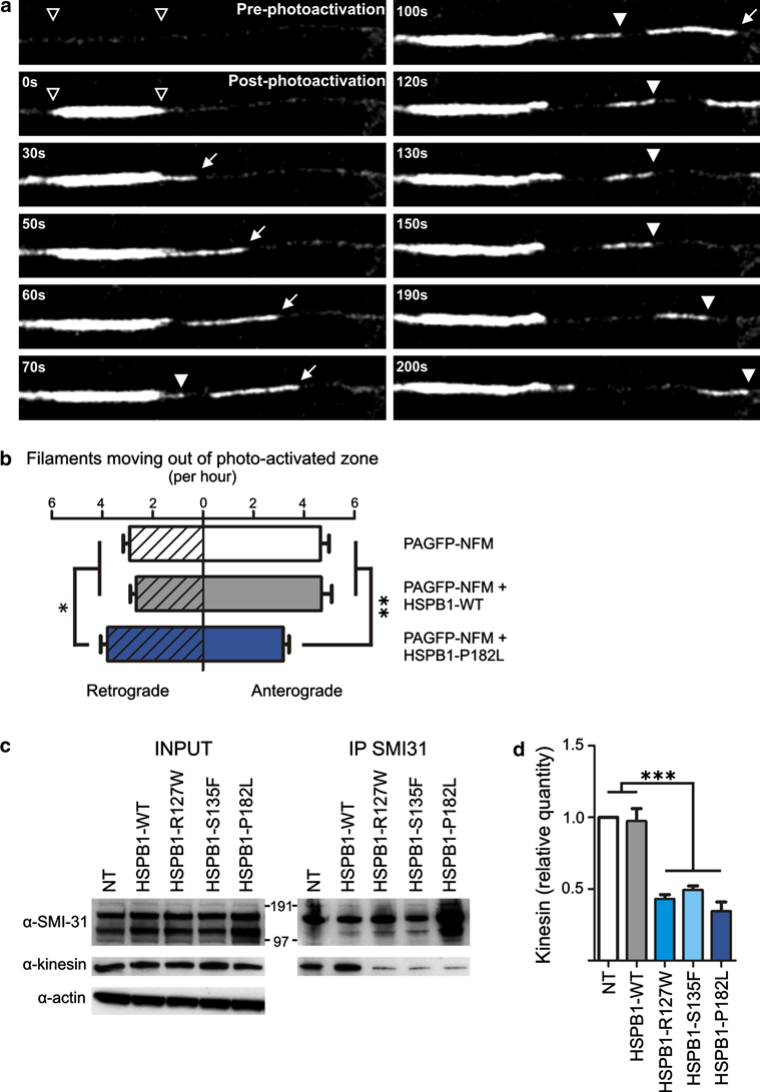
Neurofilaments move less in the anterograde direction and bind less to kinesin in mutant HSPB1 SH-SY5Y cells. **a** Live cell imaging experiments showing the movement of PAGFP-NFM. The first frame is an image of an axonal neurite before photoactivation of PAGFP-NFM. The second frame shows an image of the photoactivated zone (indicated by *open arrowheads*). The following frames show photoactivated filaments that moved out of the photoactivated zone. Pictures are taken 30 to 200 s after the photoactivation (the *arrow* and the *full arrowhead* indicate two separate moving filaments). **b** Quantification of the amount of filaments moving out of the activated zone in anterograde and retrograde direction (per hour), indicating a shift in the direction of NF movement in mutant (P182L) HSPB1 cells. **c** Co-IP showing the interaction between phosphorylated NFs and kinesin. Phospho-NFs were immunoprecipated with the SMI-31 antibody. **d** Relative quantification of the co-IP experiments shown in **c**, by calculating the ratio of kinesin in the IP over phosphorylated NFs. *NT* non-transduced cells; *p < 0.05; **p < 0.01; ***p < 0.001; *n* = 3

To test if mutations in HSPB1 affect axonal transport of NFs, we performed live cell imaging of axonal segments of wild-type (WT) and mutant HSPB1 cells expressing NFM coupled to a photoactivatable GFP (PAGFP-NFM) [20]. This method allowed us to visualize the photoactivated fluorescent filaments moving into non-photoactivated axonal regions (Fig. 1a). We used human neuroblastoma cells (SH-SY5Y) because these cells express all NF subunits endogenously. The SH-SY5Y cells were transduced with lentiviral vectors containing WT or mutant HSPB1 as described in “Materials and methods”. When cells expressed only PAGFP-NFM or PAGFP-NFM and WT HSPB1, the majority of filaments moved anterogradely, whereas cells expressing PAGFP-NFM and mutant HSPB1-P182L showed a higher frequency of filaments moving retrogradely, and a lower frequency of filaments moving anterogradely (Fig. 1b). To determine whether this effect was a consequence of a decrease in axonal transport rates, we compared the speed at which NFs were transported in cells expressing WT and mutant HSPB1. The NF axonal transport velocity results from rapid anterograde or retrograde movements interrupted by long periods of pause [47, 67]. We therefore evaluated NF movement velocity, pausing frequency and the frequency of switches made between retrograde and anterograde movements, but could not observe any statistical difference between cells expressing the different HSPB1 constructs (Table 1). We conclude that mutant HSPB1 decreases the frequency of anterograde transport of NFs in favor of retrograde transport.

**Table 1:** Parameters of NF axonal transport, measured through live cell imaging with a PAGFP-NFM construct

	PAGFP-NFM	HSPB1-WT	HSPB1-P182L
Pausing time (%)	54.32 ± 3.59	56.71 ± 4.66	59.01 ± 3.93**
Mean velocity, pause included (μm/s)	0.099 ± 0.007	0.095 ± 0.008	0.091 ± 0.006
Mean velocity per moving second, pause excluded (μm/s)	0.166 ± 0.011	0.162 ± 0.013	0.164 ± 0.011
Frequency of directionality switches (s^−1^)	0.021 ± 0.001	0.020 ± 0.002	0.021 ± 0.001

Data are represented as mean ± SEM. Pause was defined as speed rates between −0.06 and 0.06 μm/s. To determine “mean velocity” and “mean velocity per second”, anterograde and retrograde parameters were grouped, neglecting transport direction. Switches are defined as changes in directionality of the movement

** p < 0.01

### Mutation in HSPB1 decreases neurofilament binding to kinesin

Anterograde transport of NFs along the axon is mediated by the motor protein kinesin, while dynein allows both anterograde and retrograde transport [64]. To determine whether the altered axonal transport in mutant HSPB1 SH-SY5Y cells is caused by a deficit in the interaction between NFs and their motor proteins, we co-immunoprecipitated (co-IP) phosphorylated NFs and kinesin or p150glued, a component of the dynactin–dynein complex [17, 49]. We used the SMI-31 antibody that recognizes phosphorylated NFs that are able to interact with kinesin [66]. The SMI-31 antibody usually detects four separate signals in the SH-SY5Y cells, of which the upper three bands probably correspond to different phospho-forms of NFH and the single lower band corresponds to phospho-NFM. The interaction between NFs and kinesin was significantly reduced in all cell lines expressing a mutant HSPB1 construct (Fig. 1c, d). In contrast, HSPB1 mutations did not affect NF binding to p150glued (Supplementary Fig. 1). These results suggest that mutant HSPB1 may affect axonal transport of NF by altering the NFs–kinesin interaction.

### Mutation in HSPB1 increases NF–NF interaction

**Fig. 2:**
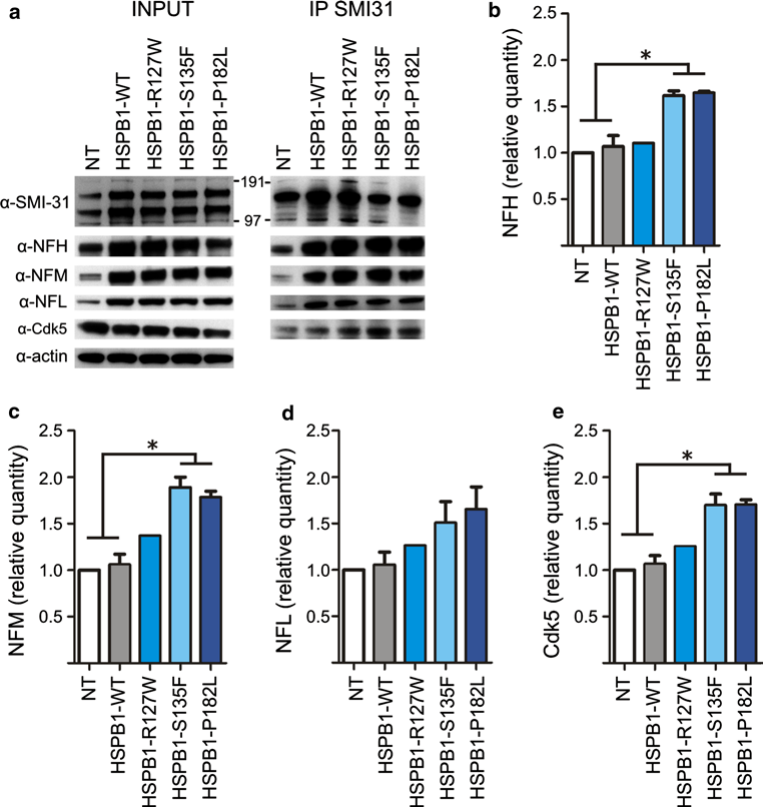
Neurofilaments in mutant HSPB1 cell lines bind more to each other and to Cdk5. **a** Co-IP experiment showing the interaction patterns between the NFs in the different cell lines, after immunoprecipitating phospho-NFs with the SMI-31 antibody. **b**–**e** Relative quantification of the co-IP experiments with specific antibodies against NFH (**b**), NFM (**c**), NFL (**d**) and Cdk5 (**e**). *NT* non-transduced cells; *p < 0.05; *n* = 3

The axonal transport of neurofilaments is mediated by continued shuttling between mobile and stationary states (reviewed in [29]). In the stationary phase, bundles of NFs are closely apposed, phosphorylated NFs that gained higher binding affinity for each other [17, 19]. Hence, the decreased interaction between the NFs and kinesin, observed in cells with mutant HSPB1, is likely to coincide with an increase in NF bundling. Therefore, we compared NF–NF interactions in cells expressing WT and mutant HSPB1. By co-IP we observed an increased interaction between the NFs in mutant HSPB1 cell lines compared to non-transduced or HSPB1-WT controls (Fig. 2a–d).

**Fig. 3:**
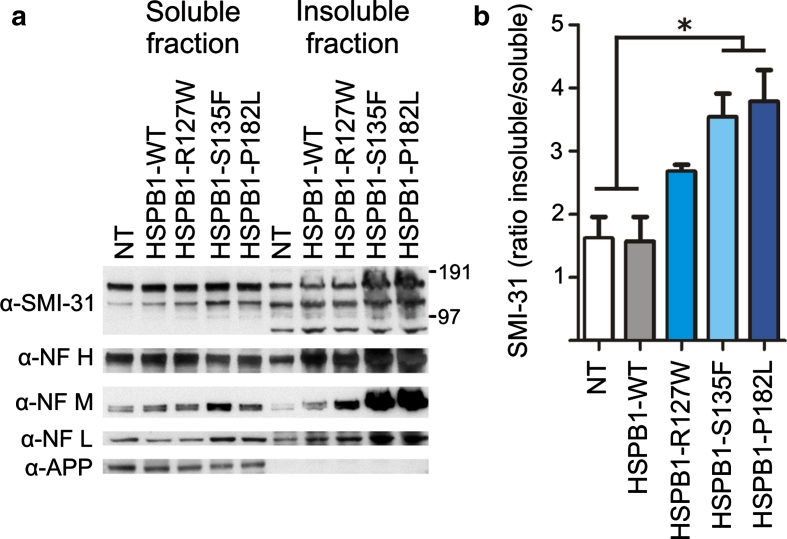
Neurofilaments in mutant HSPB1 cell lines are more Triton-X 100 insoluble. **a** Western blot of Triton-X 100-soluble and -insoluble fractions of mutant HSPB1 and controls cells. APP was used as Triton-X 100-soluble positive control. **b** Quantitative analysis of the Triton-X 100 experiments shown in **a**. The ratio insoluble/soluble fractions was calculated for each cell line and for each NF specific antibody, but is only depicted for the SMI-31 antibody. *NT* non-transduced cells; *p < 0.05; *n* = 3

**Fig. 4:**
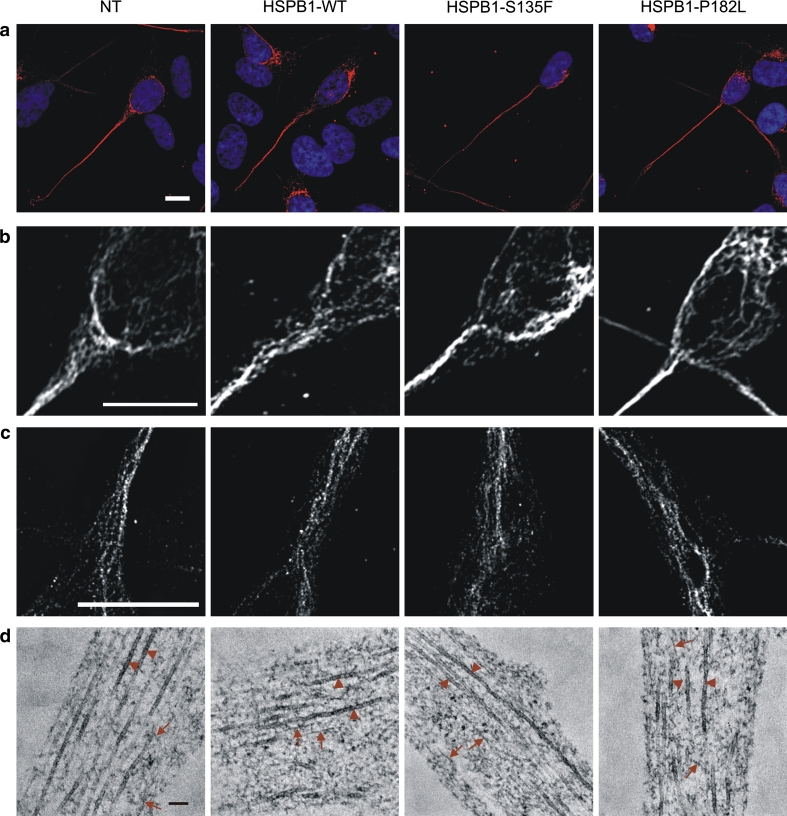
Mutant HSPB1 does not affect neurofilament network formation or neurofilament bundling. No differences in the organization or bundling of the NF network were observed in SH-SY5Y cells stably expressing WT or mutant HSPB1 construct, by using different microscopy techniques. **a**–**c** Cells immunostained for NFH were imaged with confocal laser scanning microscopy followed by 3D iterative deconvolution (**a**, **b**) and by superresolution structured illumination microscopy (**c**). **d** At the ultrastructural level, no differences in intermediate filament distribution could be observed between the cells expressing the different constructs, by using transmission electron microscopy. No clear occurrence of NF bundles was present after evaluating at least 30 neurites per genotype. The *scale bar* is 10 μm in **a**–**c** and 100 nm in **d**. *NT* non-transduced cells; *arrowheads* microtubules, *arrows* intermediate filaments

As NF bundles form polymers that are insoluble compared to their monomeric counterparts, we assessed NF solubility using the Triton-X 100 detergent. In cells stably expressing mutant HSPB1-S135F and HSPB1-P182L, the phosphorylated NFs were more retained in the insoluble pellet fraction compared to control cells (Fig. 3a–b), suggesting that NFs could be trapped in NF bundles or aggregates. We tested this hypothesis by analyzing NF morphology in the different cell lines by staining for NFH, often used as a neuronal marker (Fig. 4a). However, we could detect neither NF-positive aggregates nor any significant morphological differences between WT and mutant HSPB1 expressing cells. The NFs were present in a filamentous network independently of the evaluated HSPB1 genotype, and no differences in NF bundling were detected (Fig. 4b–c). To evaluate the intermediate filaments at the ultrastructural level, we next performed transmission electron microscopy on the axonal neurites of the SH-SY5Y cells. However, we were unable to detect differences in the intermediate filament distribution between WT and mutant HSPB1 cells (Fig. 4d).

### Mutant HSPB1 increases neurofilament phosphorylation

**Fig. 5:**
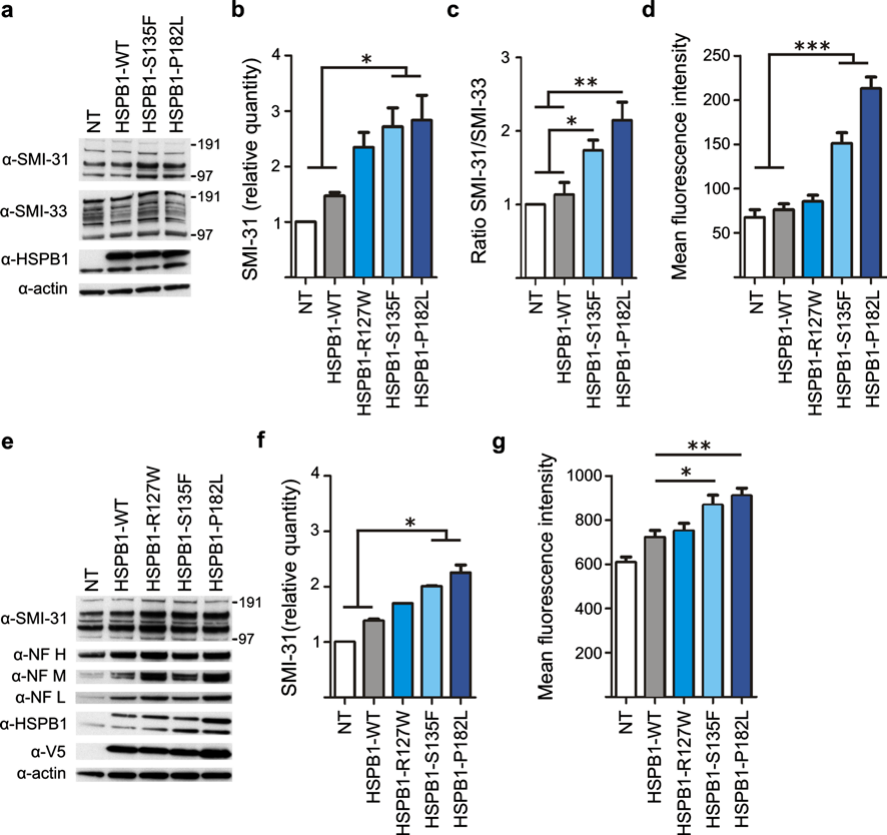
Mutant HSPB1 cell lines show hyperphosphorylated neurofilaments. **a** Western blot experiment showing phosphorylated (SMI-31) and non-phosphorylated NFs (SMI-33) from SH-SY5Y cells stably transduced with HSPB1. **b** Relative quantification of the phosphorylated NF signal intensities of HSPB1 transduced and non-transduced (NT) cells (*n* = 3). **c** Ratio of phospho- versus non-phospho-NFs after quantification of the relative band intensities. **d** Mean fluorescent intensity after immunostaining of phosphorylated NF on non-differentiated SH-SY5Y cells [non-transduced (number of cells = 179), HSPB1-WT (*n* = 234), HSPB1-R127W (*n* = 173), HSPB1-S135F (*n* = 204) and HSPB1-P182L (*n* = 220) from 4 independent experiments]. **e** Western blot experiment showing phospho-NFs and non-phoshorylated NFs in differentiated SH-SY5Y cells. The anti-V5 antibody shows the expression of the HSPB1-V5 constructs. **f** Relative quantification of the phosphorylated NF signal intensities after neuronal differentiation with retinoic acid (*n* = 2). **g** Quantification of the mean fluorescent intensity of phosphorylated NF in differentiated SH-SY5Y cells [non-transduced (*n* = 122), HSPB1-WT (*n* = 134), HSPB1-R127W (*n* = 143), HSPB1-S135F (*n* = 131) and HSPB1-P182L (*n* = 136) from 3 independent experiments). *NT* non-transduced cells; *p < 0.05; **p < 0.01; ***p < 0.001

Thus far, we have demonstrated that in SH-SY5Y cell lines transduced with CMT-associated HSPB1 mutants, NFs undergo less anterograde transport and interact less with kinesin, but bind more to each other. Interestingly, each of these properties can be regulated by the same event: phosphorylation of the large NF subunits [1, 70, 71]. Through the numerous KSP repeat motifs in their long C-terminal tail domain, NFH and NFM can be highly phosphorylated and play a crucial role in NFs properties [32, 59]. We investigated the phosphorylation status of NFs in SH-SY5Y cells expressing WT or mutant HSPB1 using the SMI-31 antibody. When cells were transduced with mutant forms of HSPB1, the level of phosphorylated neurofilament was slightly but significantly increased (Fig. 5a–b). The same effects were observed when cells were subjected to neuronal differentiation with retinoic acid (Fig. 5e, f). Subsequently, the undifferentiated and differentiated SH-SY5Y cell lines were immunostained for phosphorylated NFs, and quantification of the mean fluorescence intensity confirmed the significant increase in phosphorylated NFs in the mutant HSPB1-S135F and HSPB1-P182L cell lines (Fig. 5d–g). It is important to note that the transduction of cells with an HSPB1 construct (WT or mutant) increased the expression of the NFs, compared to non-transduced control cells. We observed a positive correlation between the protein expression levels of HSPB1 and non-phosphorylated NFs (Pearson correlation for linear measures: R = 0.6084; *p* value: 0.0358; *n* = 12). This increase reflects a more general effect of HSPB1 on the expression level of the NFs. However, when calculating the ratio of phosphorylated versus non-phosphorylated NFs, we found that the increase in phospho-NFs was independent of the increase in non-phosphorylated NFs (Fig. 5c). There was no positive correlation between the protein expression level of HSPB1 and phosphorylated NFs. We therefore conclude that mutant HSPB1 induces an increase in phospho-NFs and this increase is not attributable to HSPB1 levels.

### Increased NF phosphorylation in HSPB1 mutant cells is mediated by Cdk5

**Fig. 6:**
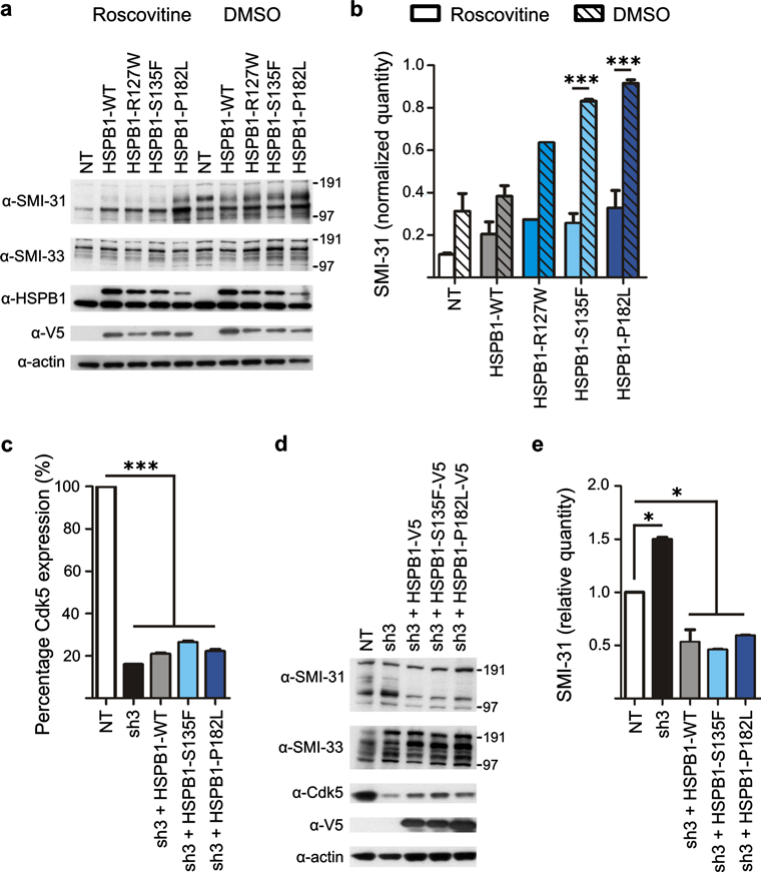
Inhibition and specific knockdown of Cdk5 restore the NF phosphorylation status in mutant HSPB1 cell lines. **a** Western blot showing the NF phosphorylation in SH-SY5Ycells incubated for 24 h with 10 μM roscovitine (Cdk5/p35 inhibitor) or dimethyl sulfoxide (DMSO) as a control (*n* = 3). **b** Quantification of the band intensities of the experiment shown in **a**. The data were normalized against actin (*n* = 3). **c** Quantification of signal intensities to assess the residual protein level of Cdk5 in the Cdk5 knockdown cell line sh3 (*n* = 3). The percentage of Cdk5 protein was calculated with the non-transduced cells as control, as the signal intensity of the latter was set to 100 %. **d** Western blot experiment showing the NF phosphorylation in WT and mutant HSPB1 cell lines, where Cdk5 was silenced by shRNA. The anti-Cdk5 antibody shows the level of Cdk5 knockdown, whereas anti-V5 antibody shows the expression of the HSPB1-V5 constructs. **e** Quantitative analysis of the level of phospho-NF in WT and mutant HSPB1 cell lines, after Cdk5 silencing. *NT* non-transduced cells; *p < 0.05; ***p < 0.001; *n* = 3

Because of the observed effects of mutant HSPB1 on the phosphorylation of NFs, we searched for the protein kinase responsible for the increased NF phosphorylation in mutant HSPB1 cells. Five proline-directed kinases have previously been shown to be able to phosphorylate the KSP repeat motifs in the C-tail domains of NFM and NFH: ERK1/2, p38, JNK, GSK3β and Cdk5 [3, 9, 15, 65]. Here, we selectively inhibited the activity of these five kinases and found that only roscovitine, a Cdk5/p35 inhibitor, prevented NF hyperphosphorylation in mutant HSPB1 cell lines. The data for roscovitine is shown in Fig. 6a, b. We confirmed these results in a second experiment where Cdk5 was silenced using a specific shRNA construct (sh3) that knocked down the expression of Cdk5 to 20 % in WT and mutant HSPB1 cell lines (Fig. 6c–e).

**Fig. 7:**
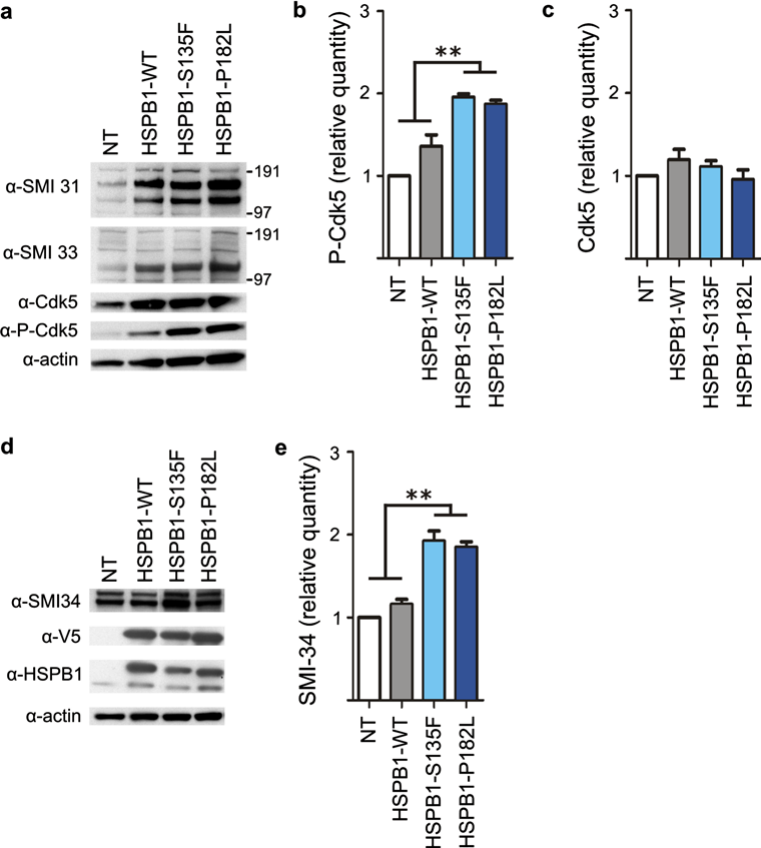
Cdk5/p35 is more active in mutant HSPB1 cell lines. **a** Western blot showing the Tyr15 phosphorylation level of Cdk5 (P-Cdk5) in non-transduced, WT and mutant HSPB1 SH-SY5Y cells. The total Cdk5 levels were detected with a non-phospho-specific Cdk5 antibody (*n* = 3). **b** Relative quantification of the phospho-Y15-Cdk5 signals. **c** Relative quantification of the non-phospho-Cdk5 signals. **d** Western blot experiment showing the phosphorylation level of NFs recognized by the SMI34 antibody. **e** Relative band quantification of signal intensities for the SMI-34 antibody shown in d. *p < 0.05; **p < 0.01; *n* = 3

Two major phosphorylation sites in Cdk5 have been shown to regulate its activity. Phosphorylation of Thr14 inhibits the activity of Cdk5, while phosphorylation of Tyr15 increases its activity [23]. To determine whether HSPB1 mutations could affect Cdk5 activity, we compared Cdk5 phosphorylation in WT and mutant HSPB1 cell lines. We found an increased phosphorylation of Cdk5 at the Tyr15 site in mutant HSPB1 cell lines (S135F and P182L), compared to controls (Fig. 7a–b), while the level of the Cdk5 protein was unaltered (Fig. 7c). We conclude that Cdk5 activity is increased in HSPB1 mutants.

To further assess the role of the increased Cdk5 activity, we compared the interaction of Cdk5 with the NFs in WT and mutant HSPB1 cells. Accordingly, our results showed an increased binding of Cdk5 to the phosphorylated NFs in protein extracts from the mutant HSPB1 cell lines (Fig. 2a, e). Furthermore, the HSPB1-S135F and P182L mutant cell lines displayed an increased phosphorylation of the S/TPXH/K/R repeat motif in the C-domains of NFH and NFM, a site specifically targeted by Cdk5/p35, which can be detected by the SMI-34 antibody specifically (Fig. 7d–e). Together, these results suggest that increased phosphorylation of NFs in HSPB1 mutant neuronal cells is mediated by an increased activity of Cdk5.

### Silencing of Cdk5 restores NF–kinesin interaction and NF solubility in HSPB1 mutants

**Fig. 8:**
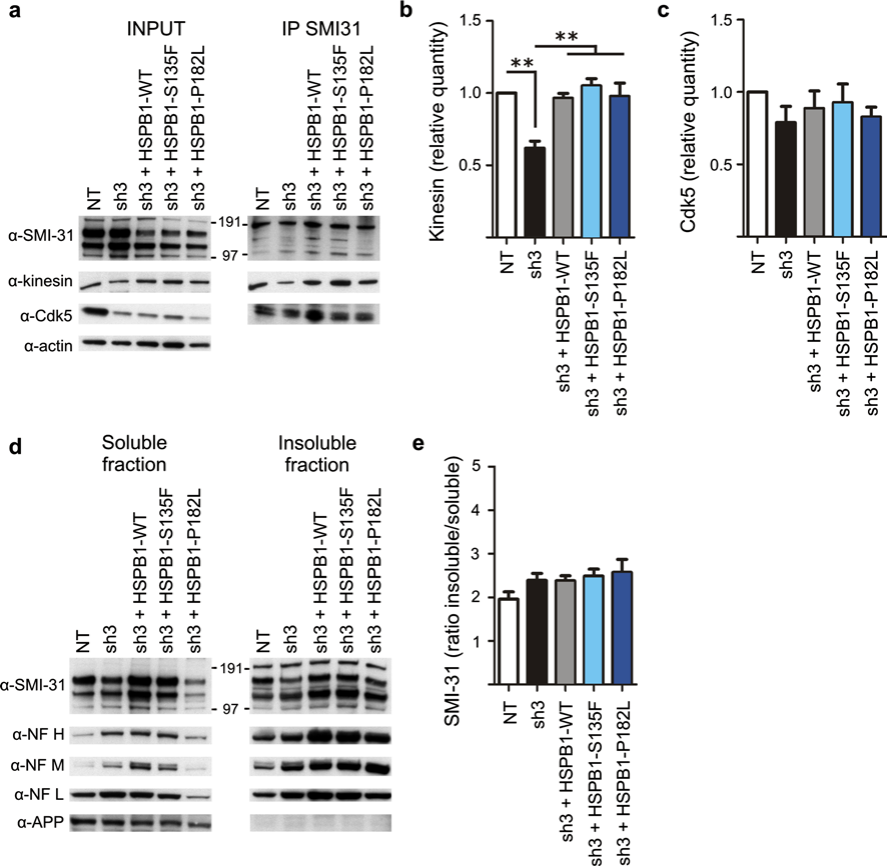
Specific knockdown of Cdk5 restores the interaction between phosphorylated neurofilaments and kinesin or Cdk5, and the neurofilament insolubility in mutant HSPB1 cell lines. **a** Repetition of the co-IP SMI-31 experiment shown in Figs. 1c and 2a, but performed with the sh3 double-transduced SH-SY5Y cell lines (*n* = 3). **b**–**c** Quantitative analysis of the co-IP experiment for kinesin (**b**) and Cdk5 (**c**). **d** Repetition of the Triton-X 100 experiment as shown in Fig. 3, but performed with the sh3 double-transduced SH-SY5Y cell lines. **e** Quantifications of the insoluble/soluble ratios of the Triton-X 100 experiments as previously described (*n* = 3). **p < 0.01

To further assess the role of Cdk5 in mutant HSPB1 cell lines, we tested if silencing of Cdk5 with shRNA could prevent the atypical NF properties: decreased binding of NFs to kinesin, increased NF-Cdk5 interaction and increased NF solubility. As expected, when Cdk5 was constitutively silenced, no difference in NF–Cdk5 interaction could be observed between WT and mutant Cdk5-silenced cells (Fig. 8a, c). Furthermore, silencing of Cdk5 also prevented the decreased binding of NFs with kinesin formerly seen in HSPB1 mutant cell lines (Fig. 8a, b). Additionally, no differences could be seen in NF solubility between WT and mutant cell lines (Fig. 8d, e). These experiments suggest that silencing Cdk5 can prevent the aberrant NF phenotype observed in cells expressing mutant HSPB1.

## Discussion

In this study, we show that CMT-causing mutations in HSPB1 reduce the anterograde axonal transport of NFs, which might be a consequence of decreased affinity of NFs for the anterograde motor protein kinesin in mutant cell lines. We demonstrated that the impaired NF–kinesin binding is in part attributable to an increase in phosphorylation of KSP sites of NFs. Although NF hyperphosphorylation was subtle and transient, we showed that in mutant cell lines, NFs displayed known characteristics of hyperphosphorylation, as their insolubility and affinity toward each other were increased. Finally, we demonstrated that these effects were at least partially mediated by the proline-directed kinase Cdk5.

Our results, showing that NF axonal transport is associated with NF phosphorylation, are in line with previous studies [37, 41]. Our time lapse imaging showed that NFs underwent both anterograde and retrograde transport interrupted by pausing periods, as previously described [47, 67]. It is reported that 73–80 % of NFs are generally in pause [47, 67], while we observed only 54–59 % of the NFs pausing. However, with our imaging method only those NFs leaving the photoactivated zone (and pausing at some point) are monitored and not those that remain in the photoactivated zone, resulting in an apparent underestimation of the NF pausing frequency.

Anterograde axonal transport of NFs is known to be mediated by kinesin [69] and we found that NF interaction with kinesin is phospho-dependent, confirming several studies [33, 37, 41, 70]. Our results suggest that the affected anterograde axonal transport observed in our mutant cell lines might play a crucial role in the pathomechanisms of CMT neuropathy.

Our results strongly suggest a central role for NFH and NFM phosphorylation in the NF atypical properties observed in HSPB1 mutants. Our strongest evidence comes from the demonstration that knocking down Cdk5 prevents NF hyperphosphorylation and interaction with kinesin. Inhibition and knockdown of Cdk5/p35 did not completely prevent NF phosphorylation, suggesting the involvement of other proline-directed kinases. The increased NF phosphorylation in cells only expressing the Cdk5 shRNA construct suggests that some compensatory mechanism by other kinases known to phosphorylate NF [51] may take place. Further support for the role of Cdk5 in NF axonal transport has been shown in literature [54]. Our study suggests a connection between the activity of Cdk5, NF hyperphosphorylation and the consequences on NF binding and dynamics in mutant HSPB1 cells. However, the intermediates in this molecular cascade are missing. Although HSPB1 and Cdk5/p35 are not known to interact with each other, they are involved in many common pathways such as apoptosis, reorganization of the cytoskeleton, cell growth and differentiation [35]. HSPB1 and Cdk5 have also been shown to be upregulated by Heat Shock Factor 2 [18, 27]. However, to our knowledge no direct or indirect interaction between the two proteins has been described. As HSPB1 is known to bind to the p38 mitogen-activated protein kinase [58], its indirect effect, through ERK1/2, on Cdk5 is a plausible hypothesis that is yet to be tested.

Although the three mutations studied here induce a decreased interaction between NFs and the motor protein kinesin, the NF–NF and NF–Cdk5 interactions were not significantly increased in the HSPB1-R127W mutant cells. This observation indicates that various mutations may differentially affect HSPB1 structure and function. Indeed, in a previous work we demonstrated differences in the binding affinity of various HSPB1 mutants according to the localization of the mutation in the protein [5]. Subsequently, it is possible that each mutation differentially affects HSPB1 post-translational modification and interaction with molecular targets, resulting in the initiation of different signaling cascades. In patients, the various mutations in the *HSPB1* gene result in differences in the severity of the disease, the involvement of the sensory and motor functions and the age of onset, which may also suggest differences in the pathomechanisms involved.

In addition to a deficit in NF axonal transport, increased Cdk5 activation and hyperphosphorylation of NFs might as well directly affect axonal degeneration in HSPB1 mutants as both events have been associated with neurodegenerative diseases [10, 42, 75]. Hyperphosphorylation of NFs is an early event observed in many neurodegenerative disorders, such as Alzheimer’s disease (AD), amyotrophic lateral sclerosis (ALS), and Parkinson’s disease (PD), for which they are used as early biomarkers [13, 14, 24, 44, 52]. Changes in the phosphorylation status of NFM and NFH have been shown to affect axonal transport and axon caliber [55], and phosphorylation of the KSP repeats in the tail domain of NFH modifies the binding properties of the NFs [56]. Hyperphosphorylation of NFs has also been linked specifically to axonal damage [45]. While normally phosphorylated NFs are found in the distal part of the axon [49, 60], hyperphosphorylated NFs are found within protein accumulations called ‘spheroids’ in the proximal axons and neuronal cell body [32, 56]. An increased amount of hyperphosphorylated NF bundles could destabilize the axonal cytoskeleton and negatively affect the axonal transport of other cargos [31, 36, 43, 68]. It was hypothesized that NF aggregates entrap kinesin and dynein motors and thus jeopardize overall axonal transport [21, 63]. However, we were unable to detect cell body modifications including NF aggregates or differences in the distribution of intermediate filaments despite the reported correlation between NF hyperphosphorylation, NF–NF interaction and bundles [37, 61]. Although significant, the NF hyperphosphorylation seen in our study might not be strong or sustained enough to support an increased bundling of NF. The apparent contradiction between the absence of aggregates in this study and the aggregates reported in a previous study using a similar cellular model [4, 74] can be explained by the level of expression of HSPB1. Indeed, the overexpression of mutant HSPB1 leads to aggregation of the protein (data not shown). While the studies previously cited used cells transiently overexpressing HSPB1, we favored a stable cell model where the transgene was expressed at a moderate level and formation of aggregates was prevented.

Another possibility is that the effect of mutant HSPB1 on NFs causes cytoskeleton dynamic deficits indirectly, through the interaction of NFs to microtubules (MTs). We recently showed that some CMT-causing HSPB1 mutations stabilized the MT network and reduced MT dynamics by binding more strongly to MTs than wild-type HSPB1 [6, 7]. On the other hand, NFs have also been shown to bind unassembled tubulin, inhibit MT polymerization and directly modulate MT assembly in the axon in vivo [12, 30, 76]. Interestingly, the interaction between NFs and tubulin was shown to be lost upon Cdk5-mediated phosphorylation of NFH [40]. The Cdk5-dependent hyperphosphorylation of NFs could therefore affect NF regulation of MT polymerization. Thus, HSPB1 mutations could reduce neuronal maintenance by contributing to global transport defects affecting the MT network directly and indirectly, through deregulation of the level of phospho-NFs. Both MT overstabilization [7] and NF hyperphosphorylation could contribute to the destabilization of the cytoskeleton, affect axonal transport and compromise the overall integrity of the axon.

Patients carrying an HSPB1 mutation are characterized by an axonal peripheral neuropathy associated with a decrease in the amplitude of the compound muscle action potentials, which is consistent with the observed decrease in the amount, density and diameter of myelinated nerve fibers [11, 38, 39, 62]. Among the few reports of biopsy of CMT2F patients, one [11] describes a single patient with an HSPB1-S135C mutation showing loss of NFs with a simultaneous increase in MT density in the sural nerve biopsy, underlining some correlation between these structures and the pathomechanisms. However, a causal link between deficits in the structure, density or function of NF and the degeneration of the peripheral axons still needs to be demonstrated.

In conclusion, we have shown that mutations in HSPB1, a stress protein with ubiquitous functions, affect NF properties, and each of these properties have a direct or indirect effect on NF axonal transport. Our present and previous works suggest that degeneration of long peripheral nerves associated with CMT results from the deleterious combination of different pathomechanisms affecting axonal transport. Importantly, our study contributes to the unraveling of common pathomechanisms linking mutations in HSPB1 and NFL to CMT diseases.

## Electronic supplementary material

Below is the link to the electronic supplementary material.

**Supplementary Figure 1: Neurofilaments in mutant HSPB1 SH-SY5Y cells do not show a difference in binding to p150glued.** a) Co-IP showing the interaction between phosphorylated NFs and p150glued. Phospho-NFs were immunoprecipated with the SMI-31 antibody. b) Relative quantification of the co-IP experiments shown in A, by calculating the ratio of p150glued in the IP over phosphorylated NFs. NT: non-transduced cells (*n* = 3). (TIFF 5196 kb)

**Supplementary Figure 2: Mutant HSPB1 does not affect neurofilament network formation or neurofilament bundling.** Additional transmission electron microscopy images confirm that no differences in intermediate filament distribution could be observed at the ultrastructural level, between WT and mutant HSPB1 SH-SY5Y cells. The scale bar is 100 nm. NT: non-transduced cells. (TIFF 20003 kb)

## References

[1] Ackerley S, Grierson AJ, Brownlees J, Thornhill P, Anderton BH, Leigh PN, Shaw CE, Miller CC (2000). Glutamate slows axonal transport of neurofilaments in transfected neurons. J Cell Biol.

[2] Ackerley S, Thornhill P, Grierson AJ, Brownlees J, Anderton BH, Leigh PN, Shaw CE, Miller CC (2003). Neurofilament heavy chain side arm phosphorylation regulates axonal transport of neurofilaments. J Cell Biol.

[3] Ackerley S, Grierson AJ, Banner S, Perkinton MS, Brownlees J, Byers HL, Ward M, Thornhill P, Hussain K, Waby JS, Anderton BH, Cooper JD, Dingwall C, Leigh PN, Shaw CE, Miller CC (2004). p38alpha stress-activated protein kinase phosphorylates neurofilaments and is associated with neurofilament pathology in amyotrophic lateral sclerosis. Mol Cell Neurosci.

[4] Ackerley S, James PA, Kalli A, French S, Davies KE, Talbot K (2006). A mutation in the small heat-shock protein HSPB1 leading to distal hereditary motor neuronopathy disrupts neurofilament assembly and the axonal transport of specific cellular cargoes. Hum Mol Genet.

[5] Almeida-Souza L, Goethals S, De Winter, V, Dierick I, Gallardo R, Van Durme J, Irobi J, Gettemans J, Rousseau F, Schymkowitz J, Timmerman V, Janssens S (2010) Increased monomerization of mutant HSPB1 leads to protein hyperactivity in Charcot–Marie–Tooth neuropathy. J Biol Chem 285:12778–12786 10.1074/jbc.M109.082644 PMC2857091 20178975

[6] Almeida-Souza L, Timmerman V, Janssens S (2011). Microtubule dynamics in the peripheral nervous system: a matter of balance. BioArchitecture.

[7] Almeida-Souza L, Asselbergh B, d’Ydewalle C, Moonens K, Goethals S, De Winter V, Azmi A, Irobi J, Timmermans JP, Gevaert K, Remaut H, Van den Bosch L, Timmerman V, Janssens S (2011). Small heat-shock protein HSPB1 mutants stabilize microtubules in Charcot–Marie–Tooth neuropathy. J Neurosci.

[8] Ammer H, Schulz R (1994). Retinoic acid-induced differentiation of human neuroblastoma SH-SY5Y cells is associated with changes in the abundance of G proteins. J Neurochem.

[9] Bajaj NP, Miller CC (1997). Phosphorylation of neurofilament heavy-chain side-arm fragments by cyclin-dependent kinase-5 and glycogen synthase kinase-3alpha in transfected cells. J Neurochem.

[10] Bajaj NP, al-Sarraj ST, Leigh PN, Anderson V, Miller CC (1999) Cyclin dependent kinase-5 (CDK-5) phosphorylates neurofilament heavy (NF-H) chain to generate epitopes for antibodies that label neurofilament accumulations in amyotrophic lateral sclerosis (ALS) and is present in affected motor neurones in ALS. Prog Neuropsychopharmacol Biol Psychiatry 23:833–850 10.1016/s0278-5846(99)00044-5 10509378

[11] Benedetti S, Previtali SC, Coviello S, Scarlato M, Cerri F, Di PE, Piantoni L, Spiga I, Fazio R, Riva N, Natali Sora MG, Dacci P, Malaguti MC, Munerati E, Grimaldi LM, Marrosu MG, De Pellegrin M, Ferrari M, Comi G, Quattrini A, Bolino A (2010). Analyzing histopathological features of rare Charcot–Marie–Tooth neuropathies to unravel their pathogenesis. Arch Neurol.

[12] Bocquet A, Berges R, Frank R, Robert P, Peterson AC, Eyer J (2009). Neurofilaments bind tubulin and modulate its polymerization. J Neurosci.

[13] Brettschneider J, Petzold A, Junker A, Tumani H (2006). Axonal damage markers in the cerebrospinal fluid of patients with clinically isolated syndrome improve predicting conversion to definite multiple sclerosis. Mult Scler.

[14] Brettschneider J, Petzold A, Schottle D, Claus A, Riepe M, Tumani H (2006). The neurofilament heavy chain (NfH) in the cerebrospinal fluid diagnosis of Alzheimer’s disease. Dement Geriatr Cogn Disord.

[15] Brownlees J, Yates A, Bajaj NP, Davis D, Anderton BH, Leigh PN, Shaw CE, Miller CC (2000). Phosphorylation of neurofilament heavy chain side-arms by stress activated protein kinase-1b/Jun N-terminal kinase-3. J Cell Sci.

[16] Chan WK, Dickerson A, Ortiz D, Pimenta AF, Moran CM, Motil J, Snyder SJ, Malik K, Pant HC, Shea TB (2004). Mitogen-activated protein kinase regulates neurofilament axonal transport. J Cell Sci.

[17] Chan WK, Yabe JT, Pimenta AF, Ortiz D, Shea TB (2005). Neurofilaments can undergo axonal transport and cytoskeletal incorporation in a discontinuous manner. Cell Motil Cytoskeleton.

[18] Chang Y, Ostling P, Akerfelt M, Trouillet D, Rallu M, Gitton Y, El FR, Fardeau V, Le Crom S, Morange M, Sistonen L, Mezger V (2006). Role of heat-shock factor 2 in cerebral cortex formation and as a regulator of p35 expression. Genes Dev.

[19] Chen J, Nakata T, Zhang Z, Hirokawa N (2000). The C-terminal tail domain of neurofilament protein-H (NF-H) forms the crossbridges and regulates neurofilament bundle formation. J Cell Sci.

[20] Colakoglu G, Brown A (2009). Intermediate filaments exchange subunits along their length and elongate by end-to-end annealing. J Cell Biol.

[21] Collard JF, Cote F, Julien JP (1995). Defective axonal transport in a transgenic mouse model of amyotrophic lateral sclerosis. Nature.

[22] d’Ydewalle C, Krishnan J, Chiheb DM, Van Damme P, Irobi J, Kozikowski AP, Vanden Berghe P, Timmerman V, Robberecht W, Van den Bosch L (2011). HDAC6 inhibitors reverse axonal loss in a mouse model of mutant HSPB1-induced Charcot–Marie–Tooth disease. Nat Med.

[23] Dhavan R, Tsai LH (2001). A decade of CDK5. Nat Rev Mol Cell Biol.

[24] Eikelenboom MJ, Uitdehaag BM, Petzold A (2011) Blood and CSF biomarker dynamics in multiple sclerosis: implications for data interpretation. Mult Scler Int 2011:823176 10.1155/2011/823176 PMC3195856 22096644

[25] Encinas M, Iglesias M, Liu Y, Wang H, Muhaisen A, Cena V, Gallego C, Comella JX (2000). Sequential treatment of SH-SY5Y cells with retinoic acid and brain-derived neurotrophic factor gives rise to fully differentiated, neurotrophic factor-dependent, human neuron-like cells. J Neurochem.

[26] Evgrafov OV, Mersiyanova IV, Irobi J, Van den Bosch L, Dierick I, Schagina O, Verpoorten N, Van Impe K, Fedotov VP, Dadali EL, Auer-Grumbach M, Wagner K, Hilton-Jones D, Talbot K, Martin J–J, Tverskaya S, Polyakov AV, Gettemans J, Robberecht W, De Jonghe P, Timmerman V (2004). Mutant small heat-shock protein 27 causes axonal Charcot–Marie–Tooth disease and distal hereditary motor neuropathy. Nat Genet.

[27] Gao C, Negash S, Wang HS, Ledee D, Guo H, Russell P, Zelenka P (2001). Cdk5 mediates changes in morphology and promotes apoptosis of astrocytoma cells in response to heat shock. J Cell Sci.

[28] Haslbeck M, Buchner J (2002). Chaperone function of sHsps. Prog Mol Subcell Biol.

[29] Holmgren A, Bouhy D, Timmerman V (2012). Neurofilament phosphorylation and their proline-directed kinases in health and disease. J Peripher Nerv Sys.

[30] Jacomy H, Zhu Q, Couillard-Despres S, Beaulieu JM, Julien JP (1999). Disruption of type IV intermediate filament network in mice lacking the neurofilament medium and heavy subunits. J Neurochem.

[31] Julien JP (1997). Neurofilaments and motor neuron disease. Trends Cell Biol.

[32] Julien J-P (1999). Neurofilament functions in health and disease. Curr Opin Neurobiol.

[33] Jung C, Lee S, Ortiz D, Zhu Q, Julien JP, Shea TB (2005). The high and middle molecular weight neurofilament subunits regulate the association of neurofilaments with kinesin: inhibition by phosphorylation of the high molecular weight subunit. Brain Res Mol Brain Res.

[34] Kappe G, Franck E, Verschuure P, Boelens WC, Leunissen JA, De Jong WW (2003). The human genome encodes 10 alpha-crystallin-related small heat shock proteins: HspB1-10. Cell Stress Chaperones.

[35] Lalioti V, Pulido D, Sandoval IV (2010). Cdk5, the multifunctional surveyor. Cell Cycle.

[36] LaMonte BH, Wallace KE, Holloway BA, Shelly SS, Ascano J, Tokito M, Van Winkle T, Howland DS, Holzbaur EL (2002). Disruption of dynein/dynactin inhibits axonal transport in motor neurons causing late-onset progressive degeneration. Neuron.

[37] Lee S, Sunil N, Shea TB (2011). C-terminal neurofilament phosphorylation fosters neurofilament–neurofilament associations that compete with axonal transport. Cytoskeleton (Hoboken).

[38] Luigetti M, Fabrizi GM, Madia F, Ferrarini M, Conte A, Del Grande A, Tasca G, Tonali PA, Sabatelli M (2010). A novel HSPB1 mutation in an Italian patient with CMT2/dHMN phenotype. J Neurol Sci.

[39] Mandich P, Grandis M, Varese A, Geroldi A, Acquaviva M, Ciotti P, Gulli R, Doria-Lamba L, Fabrizi GM, Giribaldi G, Pizzuti A, Schenone A, Bellone E (2010). Severe neuropathy after diphtheria–tetanus–pertussis vaccination in a child carrying a novel frame-shift mutation in the small heat-shock protein 27 gene. J Child Neurol.

[40] Miyasaka H, Okabe S, Ishiguro K, Uchida T, Hirokawa N (1993). Interaction of the tail domain of high molecular weight subunits of neurofilaments with the COOH-terminal region of tubulin and its regulation by tau protein kinase II. J Biol Chem.

[41] Motil J, Chan WK, Dubey M, Chaudhury P, Pimenta A, Chylinski TM, Ortiz DT, Shea TB (2006). Dynein mediates retrograde neurofilament transport within axons and anterograde delivery of NFs from perikarya into axons: regulation by multiple phosphorylation events. Cell Motil Cytoskeleton.

[42] Nguyen MD, Lariviere RC, Julien JP (2001). Deregulation of Cdk5 in a mouse model of ALS: toxicity alleviated by perikaryal neurofilament inclusions. Neuron.

[43] Perez-Olle R, Lopez-Toledano MA, Goryunov D, Cabrera-Poch N, Stefanis L, Brown K, Liem RK (2005). Mutations in the neurofilament light gene linked to Charcot–Marie–Tooth disease cause defects in transport. J Neurochem.

[44] Petzold A (2005). Neurofilament phosphoforms: surrogate markers for axonal injury, degeneration and loss. J Neurol Sci.

[45] Petzold A, Tozer DJ, Schmierer K (2011). Axonal damage in the making: neurofilament phosphorylation, proton mobility and magnetisation transfer in multiple sclerosis normal appearing white matter. Exp Neurol.

[46] Reilly MM, Murphy SM, Laura M (2011). Charcot–Marie–Tooth disease. J Peripher Nerv Syst.

[47] Roy S, Coffee P, Smith G, Liem RK, Brady ST, Black MM (2000). Neurofilaments are transported rapidly but intermittently in axons: implications for slow axonal transport. J Neurosci.

[48] Salmon P, Trono D (2006) Production and titration of lentiviral vectors. Curr Protoc Neurosci. doi:10.1002/0471142301.ns0421s37 10.1002/0471142301.ns0421s37 18428637

[49] Sanchez I, Hassinger L, Sihag RK, Cleveland DW, Mohan P, Nixon RA (2000). Local control of neurofilament accumulation during radial growth of myelinating axons in vivo. Selective role of site-specific phosphorylation. J Cell Biol.

[50] Sasaki T, Gotow T, Shiozaki M, Sakaue F, Saito T, Julien JP, Uchiyama Y, Hisanaga S (2006). Aggregate formation and phosphorylation of neurofilament-L Pro22 Charcot–Marie–Tooth disease mutants. Hum Mol Genet.

[51] Sharma P, Veeranna Sharma M, Amin ND, Sihag RK, Grant P, Ahn N, Kulkarni AB, Pant HC (2002). Phosphorylation of MEK1 by cdk5/p35 down-regulates the mitogen-activated protein kinase pathway. J Biol Chem.

[52] Shaw G, Yang C, Ellis R, Anderson K, Parker MJ, Scheff S, Pike B, Anderson DK, Howland DR (2005). Hyperphosphorylated neurofilament NF-H is a serum biomarker of axonal injury. Biochem Biophys Res Commun.

[53] Shea TB, Beermann ML (1993). Evidence that the monoclonal antibodies SMI-31 and SMI-34 recognize different phosphorylation-dependent epitopes of the murine high molecular mass neurofilament subunit. J Neuroimmunol.

[54] Shea TB, Yabe JT, Ortiz D, Pimenta A, Loomis P, Goldman RD, Amin N, Pant HC (2004). Cdk5 regulates axonal transport and phosphorylation of neurofilaments in cultured neurons. J Cell Sci.

[55] Shea TB, Chan WK (2008). Regulation of neurofilament dynamics by phosphorylation. Eur J Neurosci.

[56] Shea TB, Chan WK, Kushkuley J, Lee S (2009). Organizational dynamics, functions, and pathobiological dysfunctions of neurofilaments. Results Probl Cell Differ.

[57] Shetty KT, Link WT, Pant HC (1993). cdc2-like kinase from rat spinal cord specifically phosphorylates KSPXK motifs in neurofilament proteins: isolation and characterization. Proc Natl Acad Sci USA.

[58] Shin JK, Jeong YT, Jo HC, Kang MY, Chang IS, Baek JC, Park JK, Lee SA, Lee JH, Choi WS, Paik WY (2009). Increased interaction between heat shock protein 27 and mitogen-activated protein kinase (p38 and extracellular signal-regulated kinase) in pre-eclamptic placentas. J Obstet Gynaecol Res.

[59] Sihag RK, Inagaki M, Yamaguchi T, Shea TB, Pant HC (2007). Role of phosphorylation on the structural dynamics and function of types III and IV intermediate filaments. Exp Cell Res.

[60] Sternberger LA, Sternberger NH (1983). Monoclonal antibodies distinguish phosphorylated and nonphosphorylated forms of neurofilaments in situ. Proc Natl Acad Sci USA.

[61] Sunil N, Lee S, Shea TB (2012). Interference with kinesin-based anterograde neurofilament axonal transport increases neurofilament–neurofilament bundling. Cytoskeleton (Hoboken).

[62] Tang B, Liu X, Zhao G, Luo W, Xia K, Pan Q, Cai F, Hu Z, Zhang C, Chen B, Zhang F, Shen L, Zhang R, Jiang H (2005). Mutation analysis of the small heat shock protein 27 gene in Chinese patients with Charcot–Marie–Tooth disease. Arch Neurol.

[63] Toyoshima I, Kato K, Sugawara M, Wada C, Masamune O (1998). Kinesin accumulation in chick spinal axonal swellings with beta, beta’-iminodipropionitrile (IDPN) intoxication. Neurosci Lett.

[64] Uchida A, Alami NH, Brown A (2009). Tight functional coupling of kinesin-1A and dynein motors in the bidirectional transport of neurofilaments. Mol Biol Cell.

[65] Veeranna Amin ND, Ahn NG, Jaffe H, Winters CA, Grant P, Pant HC (1998). Mitogen-activated protein kinases (Erk1,2) phosphorylate Lys–Ser–Pro (KSP) repeats in neurofilament proteins NF-H and NF-M. J Neurosci.

[66] Veeranna, Lee JH, Pareek TK, Jaffee H, Boland B, Vinod KY, Amin N, Kulkarni AB, Pant HC, Nixon RA (2008). Neurofilament tail phosphorylation: identity of the RT-97 phosphoepitope and regulation in neurons by cross-talk among proline-directed kinases. J Neurochem.

[67] Wang L, Ho CL, Sun D, Liem RK, Brown A (2000). Rapid movement of axonal neurofilaments interrupted by prolonged pauses. Nat Cell Biol.

[68] Xia CH, Roberts EA, Her LS, Liu X, Williams DS, Cleveland DW, Goldstein LS (2003). Abnormal neurofilament transport caused by targeted disruption of neuronal kinesin heavy chain KIF5A. J Cell Biol.

[69] Yabe JT, Pimenta A, Shea TB (1999). Kinesin-mediated transport of neurofilament protein oligomers in growing axons. J Cell Sci.

[70] Yabe JT, Jung C, Chan WK, Shea TB (2000). Phospho-dependent association of neurofilament proteins with kinesin in situ. Cell Motil Cytoskeleton.

[71] Yabe JT, Chylinski T, Wang FS, Pimenta A, Kattar SD, Linsley MD, Chan WK, Shea TB (2001). Neurofilaments consist of distinct populations that can be distinguished by C-terminal phosphorylation, bundling, and axonal transport rate in growing axonal neurites. J Neurosci.

[72] Yates DM, Manser C, De Vos KJ, Shaw CE, McLoughlin DM, Miller CC (2009). Neurofilament subunit (NFL) head domain phosphorylation regulates axonal transport of neurofilaments. Eur J Cell Biol.

[73] Yuan A, Rao MV, Sasaki T, Chen Y, Kumar A, Veeranna, Liem RK, Eyer J, Peterson AC, Julien JP, Nixon RA (2006) Alpha-internexin is structurally and functionally associated with the neurofilament triplet proteins in the mature CNS. J Neurosci 26:10006–10019 10.1523/JNEUROSCI.2580-06.2006 PMC6674481 17005864

[74] Zhai J, Lin H, Julien JP, Schlaepfer WW (2007). Disruption of neurofilament network with aggregation of light neurofilament protein: a common pathway leading to motor neuron degeneration due to Charcot–Marie–Tooth disease-linked mutations in NFL and HSPB1. Hum Mol Genet.

[75] Zhou J, Wang H, Feng Y, Chen J (2010). Increased expression of cdk5/p25 in N2a cells leads to hyperphosphorylation and impaired axonal transport of neurofilament proteins. Life Sci.

[76] Zhu Q, Couillard-Després S, Julien J-P (1997). Delayed maturation of regenerating myelinated axons in mice lacking neurofilaments. Exp Neurol.

