# Supplementary figures and images for: Charcot–Marie–Tooth causing HSPB1 mutations increase Cdk5-mediated phosphorylation of neurofilaments
Source: Acta Neuropathol. 2013 Jun 1;126(1):93–108. doi: 10.1007/s00401-013-1133-6 (PMC3963106; doi:10.1007/s00401-013-1133-6)

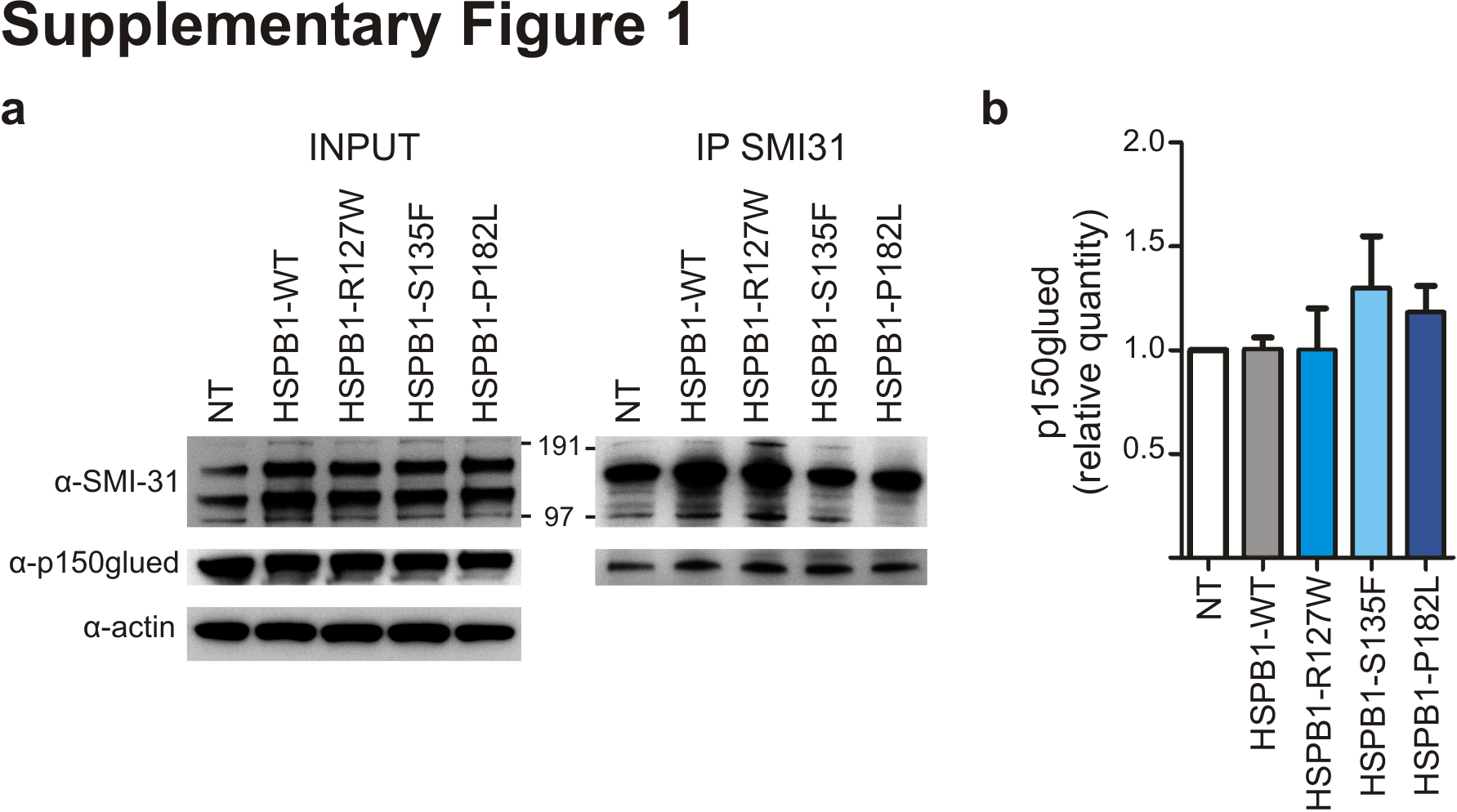

Supplement: Supplementary file 1 — Supplementary Figure 1: Neurofilaments in mutant HSPB1 SH-SY5Y cells do not show a difference in binding to p150glued. a) Co-IP showing the interaction between phosphorylated NFs and p150glued. Phospho-NFs were immunoprecipated with the SMI-31 antibody. b) Relative quantification of the co-IP experiments shown in A, by calculating the ratio of p150glued in the IP over phosphorylated NFs. NT: non-transduced cells (n = 3). (TIFF 5196 kb) [file 401_2013_1133_MOESM1_ESM.tif]

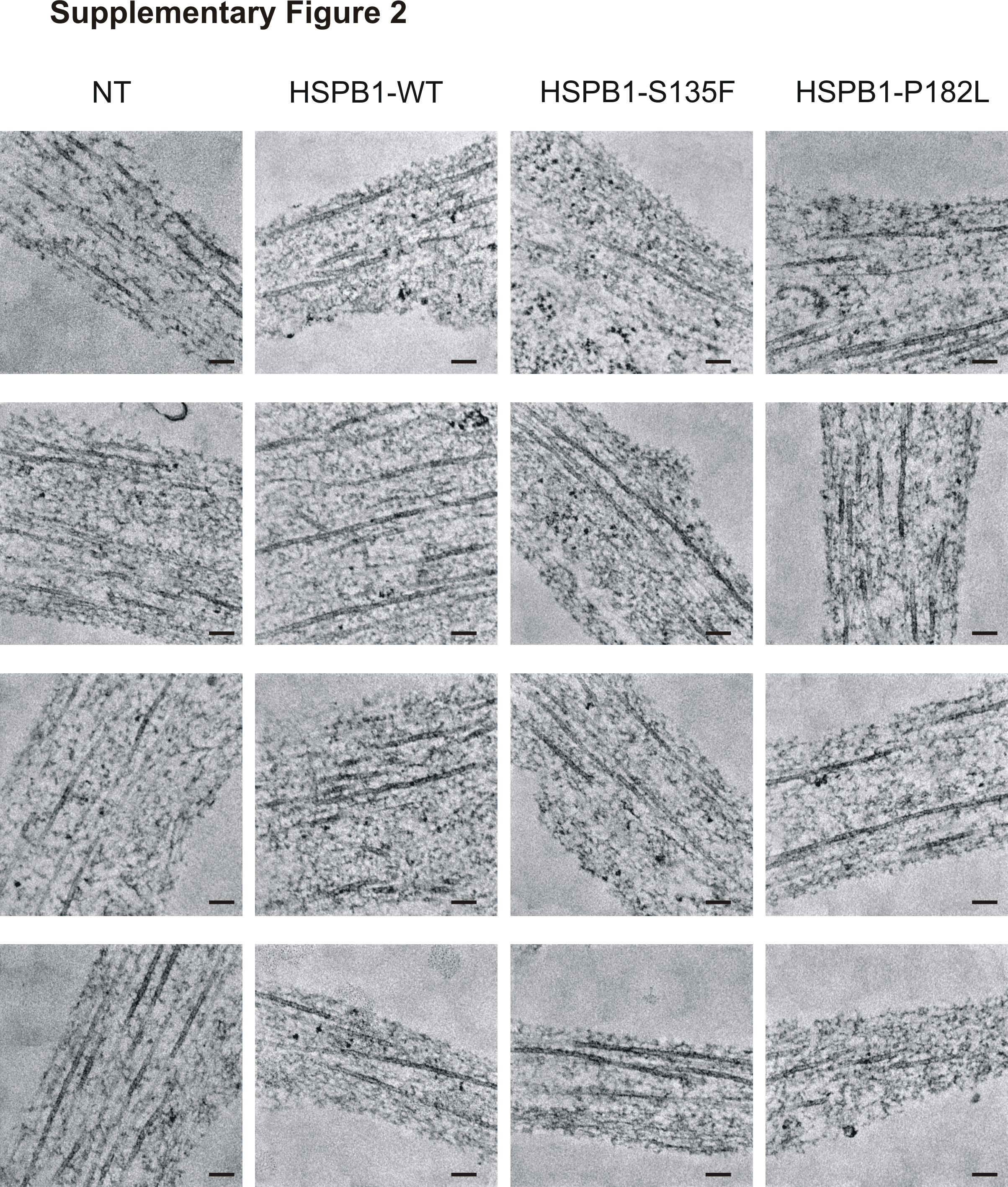

Supplement: Supplementary file 2 — Supplementary Figure 2: Mutant HSPB1 does not affect neurofilament network formation or neurofilament bundling. Additional transmission electron microscopy images confirm that no differences in intermediate filament distribution could be observed at the ultrastructural level, between WT and mutant HSPB1 SH-SY5Y cells. The scale bar is 100 nm. NT: non-transduced cells. (TIFF 20003 kb) [file 401_2013_1133_MOESM2_ESM.tif]
